# Cardiovascular Consequences of Skeletal Muscle Impairments in Breast Cancer

**DOI:** 10.3390/sports8060080

**Published:** 2020-05-31

**Authors:** Gabriel H. Zieff, Chad W. Wagoner, Craig Paterson, Patricia Pagan Lassalle, Jordan T. Lee

**Affiliations:** 1 Exercise and Sport Science, The University of North Carolina at Chapel Hill, Chapel Hill, NC 27599-8700, USA; chadwago@live.unc.edu (C.W.W.); ppagan@unc.edu (P.P.L.); jlee25@live.unc.edu (J.T.L.); 2 School of Sport and Exercise, University of Gloucestershire, Cheltenham GL2 9HW, UK; CraigPaterson@connect.glos.ac.uk

**Keywords:** skeletal muscle, breast cancer, aerobic exercise, resistance training

## Abstract

Breast cancer survivors suffer from disproportionate cardiovascular disease risk compared to age-matched controls. Beyond direct cardiotoxic effects due to treatments such as chemotherapy and radiation, breast-cancer-related reductions in skeletal muscle mass, quality and oxidative capacity may further contribute to cardiovascular disease risk in this population by limiting the ability to engage in aerobic exercise—a known promoter of cardiovascular health. Indeed, 20–30% decreases in peak oxygen consumption are commonly observed in breast cancer survivors, which are indicative of exercise intolerance. Thus, breast-cancer-related skeletal muscle damage may reduce exercise-based opportunities for cardiovascular disease risk reduction. Resistance training is a potential strategy to improve skeletal muscle health in this population, which in turn may enhance the capacity to engage in aerobic exercise and reduce cardiovascular disease risk.

## 1. Introduction

Diagnostic and therapeutic advances have drastically improved breast cancer survival rates over the last half-century [1,2], yet survivors suffer from disproportionate cardiovascular disease (CVD) risk [3,4]. Breast cancer survivors have a 1.8-fold greater risk of CVD-specific mortality compared to healthy controls [3], and CVD is now the leading cause of death among early-stage breast cancer survivors over 65 years of age [5,6]. Free radical-mediated cardiomyocyte damage and the subsequent impairment of central as well as peripheral cardiovascular function are associated with two primary cancer treatments: chemotherapy and radiation [7,8,9,10,11,12]. In the case of chemotherapeutics, free-radical damage causes dysregulation of sarcomeric proteins and myocyte necrosis [7,13]. Free radical damage resulting from radiation therapy can cause coronary vascular injury, including endothelial cell proliferation, intima-media thickening, lipid deposition, and adventitial fibrosis [11,14,15]. Ultimately, these effects can lead to, among other complications, ventricular dysfunction and heart failure [9,10,11,14,16]. Though these treatments are not used solely in breast cancer and thus elicit cardiotoxic effects in other cancer populations [17], these effects may be particularly harmful in breast cancer survivors since (i) CVDs and breast cancer share risk factors (e.g., hypercholesteremia, hypertension, obesity) [18,19,20] and (ii) most women are diagnosed with breast cancer after menopause, a period when CVD risk increases due to declining estrogen [21,22]. Additionally, many lifestyle factors such as physical inactivity and obesity (which may exist at the time of diagnosis, or develop/worsen following diagnosis and treatment) influence these shared risk factors, and may exacerbate cardiotoxic effects of treatments, thereby further contributing to CVD risk [23,24,25]. Interestingly, skeletal muscle dysfunction—including reductions in mass, quality, and oxidative capacity—is also a side-effect of cancer treatment, and has been observed in various cancer types [26,27], including breast cancer [12,28]. While skeletal muscle dysfunction may be preexisting, it can also be a direct (e.g., anthracycline-mediated skeletal muscle damage) or indirect (e.g., atrophy as a result of treatment-mediated physical inactivity) consequence of traditional treatments [12,19,28,29,30]. Skeletal muscle dysfunction may be a central component linking many therapy-related toxicities and lifestyle factors with overall CVD risk, and is potentially a unique, additional contributor to CVD risk in this population [31,32].

The detrimental effects of breast cancer treatment and lifestyle factors on cardiovascular health have been evaluated using gold standard cardiopulmonary exercise testing (CPET) assessment of peak oxygen consumption (VO_2peak_) [23,33,34]. VO_2peak_ is a global indicator of the function of the cardiovascular-muscular axis which facilitates the intake, transport, uptake, and utilization of oxygen, and is related to the capacity to perform aerobic exercise. Furthermore, VO_2peak_ is linked to CVD, disability, and mortality in healthy and clinical populations, including breast cancer [35,36,37]. Dysfunctional components of the cardiovascular–muscular axis may partially mediate the association between VO_2peak_ and CVD risk. It is also plausible that diminished VO_2peak_, an indicator of exercise intolerance, contributes to CVD risk by creating a barrier for engagement in cardio-protective behaviors such as aerobic exercise and physical activity. Of concern, breast cancer survivors typically have a VO_2peak_ 20–30% less than healthy age-matched controls [28,34,38], displaying an exercise intolerance that skeletal muscle dysfunction may be driving. Breast cancer-related treatment and lifestyle factors—which may or may not be directly related to said treatment—can lead to maladaptive changes in skeletal muscle mass, quality, function, and oxidative capacity [32,39,40]. These negative changes can contribute to reduced VO_2peak_ and promote exercise intolerance [28,32,41]. Somewhat ironically, muscular health and fitness are essential to properly engage in the very practices known to promote cardiovascular health, specifically, exercise. Therefore, compromised muscle health may not only directly place an individual at risk of poor health outcomes but may also complicate their ability to engage in the strategies that may offer them physiological protection.

In this short review, we discuss how breast cancer-related treatment and lifestyle factors may negatively impact skeletal muscle health and oxygen consumption, thereby contributing to exercise intolerance and, in turn, elevated CVD risk. More specifically, we describe how exercise intolerance as a result of breast cancer-mediated skeletal muscle damage may impede the utilization of aerobic exercise as a therapeutic, cardioprotective tool. Future considerations for researchers and clinicians are also highlighted with a particular emphasis on resistance training as a feasible strategy to combat the deleterious effects of muscle damage on CVD risk in this population.

## 2. Skeletal Muscle Damage

### 2.1. Reduced Muscle Mass

The deleterious effects of breast cancer-related treatment and lifestyle factors can negatively affect skeletal muscle size, which can contribute to functional limitations, including exercise intolerance [12,23,28]. Decreased muscle size, fiber cross-sectional area, and myocyte size have been reported in a number of cancers, including breast cancer [12,39,40]. Reductions in cross-sectional area have been shown in single fibers for both slow-twitch myosin heavy chain and fast-twitch myosin heavy chain IIa fibers [39]. Moreover, these changes seem to occur independent of weight loss [39]. Breast cancer treatments and resulting fatigue can lead to decreased physical activity [42], which likely contributes to muscle wasting as a result of disuse [12,25,43]. Chemotherapeutics may directly confer myotoxic effects which promote myofiber atrophy [12]. Specifically, oxidant stress-induced AMP-activated kinase as a result of chemotherapy can suppress protein synthesis and/or increase proteolysis [44], yet such changes may also occur independent of reactive oxygen species [12]. Reduced muscle mass has been associated with decreased aerobic capacity in rodent cancer models [45], as well as in non-cachectic and cachectic human patients with heart failure [46,47]. Muscle atrophy can decrease peripheral muscle perfusion [47], as well as promote fatigue and weakness, all of which can contribute to exercise intolerance [28].

### 2.2. Reduced Muscle Quality

Breast cancer-related treatment and lifestyle factors have been associated with impaired skeletal muscle quality, which, in turn, may contribute to exercise intolerance [32]. The most widely recognized aspect of reduced skeletal muscle quality in breast cancer is cachexia, which is characterized by intramuscular fat deposition and sarcopenia (skeletal muscle atrophy) [12,48]. Anthracycline-based chemotherapeutics have been linked to deleterious changes in the intramuscular fat to skeletal muscle ratio [49], yet these compositional shifts are also known to occur as a result of poor lifestyle factors, such as physical inactivity and obesity [50,51]. Importantly, the ratio of intramuscular fat to skeletal muscle has been inversely associated with local muscle and whole-body VO_2_ in breast cancer survivors [32] and reported to explain approximately 50% of the variability in cardiorespiratory fitness in these individuals [32]. It is not fully clear how intramuscular fat is related to VO_2_, but potential mechanisms include: (i) blood flow diversion to metabolically inactive intramuscular fat via vasodilation of adipose tissue vessels, (ii) increased distance for oxygen to travel from capillaries to muscle mitochondria, and (iii) decreased overall blood flow to tissue [32,52,53,54]. Together, these effects may limit oxygen transport, diffusion, and uptake, and thereby promote exercise intolerance [32,52,53,54].

### 2.3. Reduced Oxidative Capacity

In addition to reductions in skeletal muscle quality and mass, diminished muscular oxidative capacity has been reported among breast cancer survivors [40]. Several factors may contribute to impaired oxidative capacity in breast cancer survivors, including direct treatment-related effects (e.g., mitochondrial damage associated with treatment-mediated reactive oxygen species) and indirectly through physical inactivity [12,28]. Peripheral changes at the level of the skeletal muscle and microvasculature may explain why decreased aerobic capacity has been observed despite maintained central cardiac function in breast cancer survivors during CPETs [23,55]. Breast cancer survivors with significant reductions in VO_2peak_ may experience diminished aerobic capacity due to peripheral issues such as reduced capillary-fiber ratio and number of oxidative fibers, despite having a preserved ejection fraction [53,55]. Other potential peripheral candidates mediating the decrease in oxidative capacity seen in breast cancer include decreases in: (i) skeletal muscle blood flow, (ii) skeletal muscle oxygen diffusion, and (iii) mitochondrial content (e.g., citrate synthase) and size including within both sub-sarcolemmal and intermyofibrillar compartments, potentially resulting in impaired glycolysis and fatty acid oxidation [12,38,40,56]. Together, breast-cancer-related reductions in microvascular and metabolic function within the skeletal muscle can reduce skeletal muscle oxidative capacity and lead to exercise intolerance.

## 3. Cardiovascular and Clinical Repercussions of Skeletal Muscle Damage

The adverse changes in skeletal muscle quality, mass, and oxidative capacity contribute to a range of functional consequences and negative outcomes, including decreased quality of life (QOL) [57], fatigue [58], arm lymphedema [59], muscle weakness [29], diminished capacity to perform activities of daily living [60], and poor prognosis [61]. Impairment in skeletal muscle structure and function have also been linked to reduced VO_2peak_ and exercise intolerance [23], which may be the limitation of skeletal muscle dysfunction most tightly associated with whole-body health and CVD risk [31]. Dysfunctional components of the cardiovascular-muscular axis may partially mediate the association between VO_2peak_ and CVD risk. However, it is also possible that VO_2peak_ reductions and exercise intolerance as a result of breast cancer-related skeletal muscle damage is further contributing to CVD risk by creating a barrier for engagement in cardioprotective strategies such as aerobic exercise and physical activity. For example, leg strength has been shown to be related to both 6-min walk test [39], and VO_2peak_ [41]. Further highlighting the practical implications for exercise intolerance, one-third of breast cancer survivors may have a VO_2peak_ less than 15.4 mL/kg/min—the aerobic capacity needed for functional dependence [38]. As such, exercise intolerance may contribute to CVD risk in breast cancer survivors by limiting the potential to garner cardioprotective benefits from exercise. Ultimately, limitations at the level of the skeletal muscle may hamper breast cancer survivors’ ability to (i) engage in consistent aerobic exercise training, and/or (ii) reach a high enough exercise intensity to reap cardioprotective effects [31].

### Limited Potential for Aerobic Exercise as a Promoter of Cardiovascular Health

Various exercise modalities have been assessed as tools to promote physiological and psychological health during and following treatment in many cancers [62,63]. Aerobic exercise and resistance training have shown benefits in a variety of cancers types—including breast cancer—with improvements observed in strength, QOL, physical capacity, and mortality, among others [62,63,64,65]. While individual factors and contraindications are important to consider, exercise has generally been deemed a positive psychophysiological stimulus in many cancers. Indeed, exercise guidelines in cancer populations, including breast cancer, largely mirror guidelines for the general population, which consist of a combination of aerobic exercise and resistance training [66,67].

Indeed, structured aerobic and resistance exercise during and following breast cancer treatment seems to be mostly feasible and safe [68,69,70]. Positive psychosocial effects include improved patient reported fatigue [66], QOL [66,71], anxiety [71], and self-esteem [71]. Physiological improvements have also been reported including increased aerobic capacity (e.g., VO_2peak_) [71,72,73], muscle strength [71], and body composition [74]. Aerobic exercise in particular, is an appealing therapeutic strategy for breast cancer survivors given (i) longitudinal evidence showing strong relationships between physical activity levels and CVD outcomes in breast cancer survivors [75,76], and (ii) the potent, whole-body cardioprotective effects aerobic exercise confers in healthy and clinical populations [77,78]. Yet, limited and mixed evidence indicates beneficial cardiovascular effects of aerobic exercise in breast cancer survivors beyond improvements in VO_2peak_ (e.g., resting heart rate, systolic blood pressure). [79,80,81] The lack of evidence related to the beneficial effects on other cardiovascular markers may be related to limited studies, differences in treatment regimens, and non-uniform aerobic exercise prescriptions [19,82]. However, poor skeletal muscle health may also contribute to the heterogenous evidence pertaining to cardio-protective effects of aerobic exercise in breast cancer.

As described previously, breast cancer treatment can cause damage to skeletal muscle, which has been measured by impairment of structure (e.g., size, quality, mitochondrial content) [12,48] and function (e.g., performance, strength, functionality) [29,58]. Furthermore, skeletal muscle damage is associated with a lower aerobic capacity in clinical populations, including breast cancer survivors [32,46,47]. It is plausible that skeletal muscle impairment may prevent breast cancer survivors from participating in aerobic exercise for a long enough duration or high enough intensity to elicit physiological adaptations that are protective against CVD. Therefore, poor skeletal muscle health and function may be a prominent link between breast cancer treatment and CVD development. As such, the fitness and cardiovascular decrements as a result of age, breast cancer, and breast cancer treatment—which already place survivors at increased risk of CVD [19,83]—are even more difficult to combat if muscular health also declines. Thus, breast cancer survivors are battling a “triple-edged sword:” (i) initial CVD risk from pre-existing modifiable and non-modifiable risk factors, (ii) breast cancer treatment-mediated acquired or accelerated CVD risk, and (iii) diminished skeletal muscle health resulting in suboptimal ability to engage in exercise to combat CVD risk.

**Figure 1:**
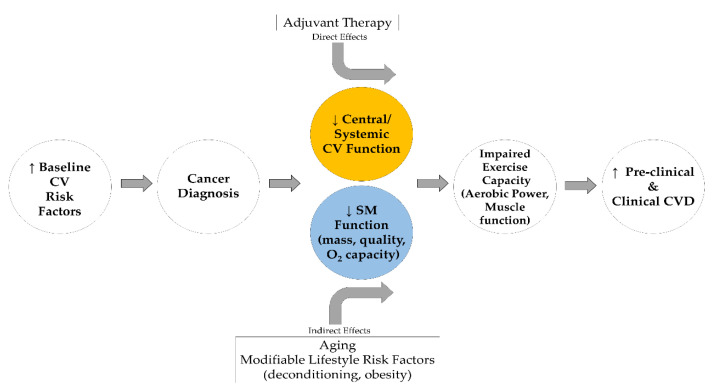
Extended “Multi-Hit” Hypothesis model adapted from Lee Jones et al. [24]. CV, Cardiovascular; O_2_, Oxidative; SM, Skeletal muscle.

Given that skeletal muscle health supports exercise and movement known to prevent CVD risk in healthy and clinical populations [84], we posit that skeletal muscle is an important factor for the prevention of CVD risk in breast cancer survivors (Figure 1). In losing the optimal health and capability of muscle, breast cancer survivors lose or decrease their ability to fight CVD risk through exercise and/or may not be able to confer enough benefit in enough time to prevent CVD development. Resistance training is a strategy to improve strength and muscle function for breast cancer survivors [85,86], which can in turn improve the ability of these individuals to engage in and sustain aerobic exercise—a known promoter of cardiovascular health.

## 4. Targeting Skeletal Muscle Health with Resistance Training

Resistance training can mitigate decreases in muscle mass, strength, aerobic capacity (VO_2peak/max_) and overall function associated with aging [87]. Furthermore, strong relationships have been reported in 1-repetition maximum scores and muscle fiber area with VO_2peak_ [88,89]. Notably, our colleagues demonstrated that as little as two weeks of resistance training can improve VO_2peak_ and force production in post-menopausal, sedentary females—the primary demographic of breast cancer survivors at high CVD risk [90]. Beneficial effects of resistance training have been reported in skeletal muscle structure and function, as well as VO_2peak,_ in clinical conditions including breast cancer [40,91,92]. As such, it is possible that resistance training in breast cancer survivors could reduce aerobic exercise intolerance by targeting skeletal muscle health, thereby improving the capacity to engage in aerobic exercise and mitigate CVD risk accrual.

Promising preliminary evidence suggests the beneficial effects of resistance training on skeletal muscle health and aerobic capacity in breast cancer survivors [91,92]. The improvements in skeletal muscle mass and strength associated with resistance training are positive adaptations in and of themselves. However, we further posit that these effects may have even greater implications by facilitating an improved ability to engage in aerobic exercise which can positively impact CVD risk. However, additional research is needed to investigate several critical gaps in the literature. Most importantly, clarification as to whether resistance training improves CVD risk in breast cancer survivors is needed. Second, if resistance training does ameliorate CVD risk in this population, efforts should be undertaken to understand if improvements in skeletal muscle health are mediating cardioprotective effects by enabling engagement in more optimal (e.g., greater duration, frequency, intensity) aerobic exercise and/or physical activity. Further work should determine optimal order/timing of exercise modalities (e.g., resistance exercise prior to traditional treatment to prime the body for aerobic exercise or combined aerobic plus resistance training following treatment).

## 5. Considerations and Recommendations

Several final considerations should be highlighted. First, one of the greatest challenges facing clinicians and researchers is understanding the complex interplay of CVD risk factors in breast cancer. This interplay has been aptly termed the “multiple hit” hypothesis, and encompasses the confluence of (i) direct cardiotoxic effects of breast cancer treatment, (ii) indirect effects of the treatment-mediated development of poor lifestyle factors (e.g., fatigue/stress-mediated physical inactivity or weight gain), and (iii) effects of pre-existing poor lifestyle factors at the time of diagnosis. [24] Understanding the etiology of CVD risk factors at the level of the individual may help shape traditional and lifestyle-based treatment strategies.

Secondly, great heterogeneity exists in (i) CVD risk accrual (or lack thereof) following cancer treatment [93], and (ii) cardiovascular outcomes (e.g., VO_2peak_, systolic blood pressure) following exercise-based therapies in clinical populations including cancer [19,82,94]. It will be challenging, but necessary, to begin to understand how and why different individuals respond uniquely to various therapies. In particular, the prospect of being able to identify “responders” versus “non-responders” may influence treatment strategies and assist in more accurate prognoses.

As alluded to previously, it will also be very difficult to determine or predict the most effective timing, order, and modality of exercise treatments relative to traditional treatments for optimizing patient health. For example, whether pre-treatment (e.g., chemotherapy, radiation) resistance training to “prime” skeletal muscles for aerobic work, followed by combined aerobic exercise and resistance training after traditional treatment is more advantageous than, say, combined exercise before and after traditional treatments remains an intriguing question. While tempting to speculate, there is likely not a “right” answer, per se. Rather, baseline characteristics (e.g., muscle strength, aerobic capacity), prior exercise history, pre-existing risk factors, cancer stage, traditional treatment plan/stage/dose, and symptom presentation following traditional treatment should weigh into individualized, and, ideally, supervised, exercise prescriptions [95]. For instance, resistance training may be the most advantageous exercise strategy to elicit high-intensity cardiac stimuli in highly deconditioned breast cancer survivors during adjunctive therapy [96]. The myriad factors that should inform exercise prescription in breast cancer survivors also highlights the need for regular interaction and coordination between oncologists and clinical exercise physiologists.

## 6. Conclusions

In conjunction with improved survival rates, breast cancer survivors are at increased risk of CVDs, including left ventricular dysfunction and heart failure [1,3,9,10]. The direct cardiotoxic effects of traditional breast cancer treatments, coupled with the indirect effects of treatment-mediated and/or pre-existing poor lifestyle factors (e.g., physical inactivity), promote elevated CVD risk [24]. Beyond breast cancer treatment-related central cardiac dysfunction, peripheral dysfunction at the level of the skeletal muscle may also contribute to CVD risk [12,61]. Specifically, decrements in skeletal muscle quality, mass, and oxidative capacity can contribute to exercise intolerance [23], which is tightly linked to CVD risk and whole-body health. [35,36,37]. Exercise intolerance limits opportunities for aerobic exercise to be used as a cardio-protective behavior in this population. Resistance training is a promising strategy to offset the negative effects of breast-cancer-related skeletal muscle dysfunction [91]. Further research is needed to (i) elucidate the mechanisms by which breast-cancer treatments and lifestyle factors impact skeletal muscle health and (ii) how skeletal muscle dysfunction may contribute to exercise intolerance, CVD risk, and whole-body health.

## References

[1] Jatoi I., Chen B.E., Anderson W.F., Rosenberg P.S. (2007). Breast cancer mortality trends in the United States according to estrogen receptor status and age at diagnosis. J. Clin. Oncol..

[2] DeSantis C., Jemal A., Ward E., Thun M.J. (2008). Temporal trends in breast cancer mortality by state and race. Cancer Causes Control.

[3] Hooning M.J., Botma A., Aleman B.M.P., Baaijens M.H.A., Bartelink H., Klijn J.G.M., Taylor C.W., van Leeuwen F.E. (2007). Long-term risk of cardiovascular disease in 10-year survivors of breast cancer. J. Natl. Cancer Inst..

[4] Jones L.M., Stoner L., Brown C., Baldi C., McLaren B. (2013). Cardiovascular disease among breast cancer survivors: The call for a clinical vascular health toolbox. Breast Cancer Res. Treat..

[5] Hanrahan E.O., Gonzalez-Angulo A.M., Giordano S.H., Rouzier R., Broglio K.R., Hortobagyi G.N., Valero V. (2007). Overall survival and cause-specific mortality of patients with stage T1a, bN0M0 breast carcinoma. J. Clin. Oncol..

[6] Patnaik J.L., Byers T., DiGuiseppi C., Dabelea D., Denberg T.D. (2011). Cardiovascular disease competes with breast cancer as the leading cause of death for older females diagnosed with breast cancer: A retrospective cohort study. Breast Cancer Res..

[7] Lim C.C., Zuppinger C., Guo X., Kuster G.M., Helmes M., Eppenberger H.M., Suter T.M., Liao R., Sawyer D.B. (2004). Anthracyclines Induce Calpain-dependent Titin Proteolysis and Necrosis in Cardiomyocytes. J. Biol. Chem..

[8] Pentassuglia L., Sawyer D.B. (2009). The role of Neuregulin-1β/ErbB signaling in the heart. Exp. Cell Res..

[9] Drafts B.C., Twomley K.M., D’Agostino R., Lawrence J., Avis N., Ellis L.R., Thohan V., Jordan J., Melin S.A., Torti F.M. (2013). Low to moderate dose anthracycline-based chemotherapy is associated with early noninvasive imaging evidence of subclinical cardiovascular disease. JACC Cardiovasc. Imaging.

[10] Harrison J., Friese C., Barton D.L., Janz N.K. (2018). Heart Failure and Long-Term Survival Among Older Women With Breast Cancer. Oncol. Nurs. Forum..

[11] Hufnagle J.J., Goyal A. (2020). Radiation Therapy Induced Cardiac Toxicity.

[12] Guigni B.A., Callahan D.M., Tourville T.W., Miller M.S., Fiske B., Voigt T., Korwin-Mihavics B., Anathy V., Dittus K., Toth M.J. (2018). Skeletal muscle atrophy and dysfunction in breast cancer patients: Role for chemotherapy-derived oxidant stress. Am. J. Physiol. Cell Physiol..

[13] Aries A., Paradis P., Lefebvre C., Schwartz R.J., Nemer M. (2004). Essential role of GATA-4 in cell survival and drug-induced cardiotoxicity. Proc. Natl. Acad. Sci. USA.

[14] Schultz-Hector S. (1992). Radiation-induced Heart Disease: Review of Experimental Data on Dose Reponse and Pathogenesis. Int. J. Radiat. Biol..

[15] Senkus-Konefka E., Jassem J. (2007). Cardiovascular effects of breast cancer radiotherapy. Cancer Treat. Rev..

[16] Pai VBNMC (2000). Cardiotoxicity of Chemotherapeutic Agents—Incidence, Treatment, and Prevention. Drug Saf..

[17] Chang H.M., Moudgil R., Scarabelli T., Okwuosa T.M., Yeh E.T. (2017). Cardiovascular Complications of Cancer Therapy: Best Practices in Diagnosis, Prevention, and Management: Part 1. JACC CardioOncol..

[18] Scott J.M., Koelwyn G.J., Hornsby W.E., Khouri M., Peppercorn J., Douglas P.S., Jones L.W. (2013). Exercise therapy as treatment for cardiovascular and oncologic disease after a diagnosis of early-stage cancer. Semin. Oncol..

[19] Scott J.M., Adams S.C., Koelwyn G.J., Jones L.W. (2016). Cardiovascular Late Effects and Exercise Treatment in Breast Cancer: Current Evidence and Future Directions. Can. J. Cardiovasc..

[20] Meijers W.C., de Boer R.A. (2019). Common risk factors for heart failure and cancer. Cardiovasc. Res..

[21] SEER Cancer Statistics Review (CSR), 1975–2017 [Internet]. https://seer.cancer.gov/csr/1975_2017/sections.html.

[22] Giordano S., Hage F.G., Xing D., Chen Y.F., Allon S., Chen C., Oparil S. (2015). Estrogen and cardiovascular disease: Is timing everything?. Am. J. Med. Sci..

[23] Beaudry R.I., Howden E.J., Foulkes S., Bigaran A., Claus P., Haykowsky M.J., Gerche A.L. (2019). Determinants of exercise intolerance in breast cancer patients prior to anthracycline chemotherapy. Physiol. Rep..

[24] Jones L.W., Haykowsky M.J., Swartz J.J., Douglas P.S., Mackey J.R. (2007). Early Breast Cancer Therapy and Cardiovascular Injury. J. Am. Coll. Cardiol..

[25] Park N.J., Chang Y., Bender C., Conley Y., Chlebowski R.T., Van Londen G.J., Foraker R., Wassertheil-Smoller S., Stefanick M.L., Kuller L.H. (2017). Cardiovascular disease and mortality after breast cancer in postmenopausal women: Results from the Women’s Health Initiative. PLoS ONE.

[26] Sturgeon K.M., Mathis K.M., Rogers C.J., Schmitz K.H., Waning D.L. (2019). Cancer- and Chemotherapy-Induced Musculoskeletal Degradation. JBMR PLUS.

[27] Gilliam L.A.A., St Clair D.K. (2011). Chemotherapy-induced weakness and fatigue in skeletal muscle: The role of oxidative stress. Antioxid. Redox Signal..

[28] Bonsignore A., Warburton D. (2013). The mechanisms responsible for exercise intolerance in early-stage breast cancer: What role does chemotherapy play?. Hong Kong Physiother. J..

[29] Bruera E., Brenneis C., Michaud M., Jackson P.I., Macdonald R.N. (1988). Muscle electrophysiology in patients with advanced breast cancer. J. Natl. Cancer Inst..

[30] Aversa Z., Costelli P., Muscaritoli M. (2017). Cancer-induced muscle wasting: Latest findings in prevention and treatment. Ther. Adv. Med. Oncol..

[31] Kirkham A.A., Beaudry R.I., Paterson D.I., Mackey J.R., Haykowsky M.J. (2019). Curing breast cancer and killing the heart: A novel model to explain elevated cardiovascular disease and mortality risk among women with early stage breast cancer. Prog. Cardiovasc. Dis..

[32] Beaudry R.I., Kirkham A.A., Thompson R.B., Grenier J.G., Mackey J.R., Haykowsky M.J. (2020). Exercise Intolerance in Anthracycline-Treated Breast Cancer Survivors: The Role of Skeletal Muscle Bioenergetics, Oxygenation, and Composition. The Oncologist.

[33] Jones L.W., Haykowsky M., Pituskin E.N., Jendzjowsky N.G., Tomczak C.R., Haennel R.G., Mackey J.R. (2007). Cardiovascular Reserve and Risk Profile of Postmenopausal Women After Chemoendocrine Therapy for Hormone Receptor Positive Operable Breast Cancer. The Oncologist.

[34] Jones L.W., Eves N.D., Mackey J.R., Peddle C.J., Haykowsky M., Joy A.A., Courneya K.S., Tankel K., Spratlin J., Reiman T. (2007). Safety and feasibility of cardiopulmonary exercise testing in patients with advanced cancer. Lung Cancer.

[35] Myers J., Prakash M., Froelicher V., Do D., Partington S., Edwin Atwood J. (2002). Exercise capacity and mortality among men referred for exercise testing. N. Engl. J. Med..

[36] Ozemek C., Laddu D.R., Lavie C.J., Claeys H., Kaminsky L.A., Ross R., Wisloff U., Arena R., Blair S.N. (2018). An Update on the Role of Cardiorespiratory Fitness, Structured Exercise and Lifestyle Physical Activity in Preventing Cardiovascular Disease and Health Risk. Prog. Cardiovasc. Dis..

[37] Peel J.B., Sui X., Adams S.A., HIbert J.R., Hardin J.W., Blair S.N. (2009). A prospective study of cardiorespiratory fitness and breast cancer mortality. Med. Sci. Sports Exerc..

[38] Jones L.W., Courneya K.S., Mackey J.R., Muss H.B., Pituskin E.N., Scott J.M., Hornsby W.E., Coan A.D., Herndon J.E., Douglas P.S. (2012). Cardiopulmonary function and age-related decline across the breast cancer: Survivorship continuum. J. Clin. Oncol..

[39] Toth M.J., Callahan D.M., Miller M.S., Tourville T.W., Hackett S.B., Couch M.E., Dittus K. (2016). Skeletal muscle fiber size and fiber type distribution in human cancer: Effects of weight loss and relationship to physical function. Clin. Nutr..

[40] Mijwel S., Cardinale D.A., Norrbom J., Chapman M., Ivarsson N., Wengström Y., Sundberg C.J., Rundqvist H. (2018). Exercise training during chemotherapy preserves skeletal muscle fiber area, capillarization, and mitochondrial content in patients with breast cancer. FASEB J..

[41] O’Donnell D.E., Webb K.A., Langer D., Elbehairy A.F., Neder J.A., Dudgeon D.J. (2016). Respiratory Factors Contributing to Exercise Intolerance in Breast Cancer Survivors: A Case-Control Study. J. Pain. Symptom. Manag..

[42] Romero S.A.D., Jones L., Bauml J.M., Li Q.S., Cohen R.B., Mao J.J. (2018). The association between fatigue and pain symptoms and decreased physical activity after cancer. Support Care Cancer.

[43] Evans W.J. (2010). Skeletal muscle loss: Cachexia, sarcopenia, and inactivity. Am. J. Clin. Nutri..

[44] White J.P., Puppa M.J., Gao S., Sato S., Welle S.L., Carson J.A. (2013). Muscle mTORC1 suppression by IL-6 during cancer cachexia: A role for AMPK. Am. J. Physiol. Endocrinol. Metab..

[45] Esau P.J., Gittemeier E.M., Opoku-Acheampong A.B., Rollins K.S., Baumfalk D.R., Poole D.C., Musch T.I., Behnke B.J., Copp S.W. (2017). Prostate cancer reduces endurance exercise capacity in association with reductions in cardiac and skeletal muscle mass in the rat. Am. J. Cancer Res..

[46] Cicoira M., Zanolla L., Franceschini L., Rossi A., Golia G., Zamboni M., Tosoni P., Zardini P. (2001). Skeletal muscle mass independently predicts peak oxygen consumption and ventilatory response during exercise in noncachectic patients with chronic heart failure. J. Am. Coll. Cardiol..

[47] Strassburg S., Springer J., Anker S.D. (2005). Muscle wasting in cardiac cachexia. Int. J. Biochem. Cell Biol..

[48] Battaglini C.L., Hackney A.C., Goodwin M.L. (2012). Cancer cachexia: Muscle physiology and exercise training. Cancers (Basel).

[49] Fearon K.C.H., Glass D.J., Guttridge D.C. (2012). Cancer cachexia: Mediators, signaling, and metabolic pathways. Cell Metab..

[50] Goodpaster B.H., Theriault R., Watkins S.C., Kelley D.E. (2000). Intramuscular lipid content is increased in obesity and decreased by weight loss. Metabolism.

[51] Manini T.M., Clark B.C., Nalls M.A., Goodpaster B.H., Ploutz-Snyder L.L., Harris T.B. (2007). Reduced physical activity increases intermuscular adipose tissue in healthy young adults. Am. J. Clin. Nutr..

[52] Goodpaster B.H., Thaete F.L., Kelley D.E. (2000). Thigh adipose tissue distribution is associated with insulin resistance in obesity and in type 2 diabetes mellitus. Am. J. Clin. Nutr..

[53] Haykowsky M.J., Kouba E.J., Brubaker P.H., Nicklas B.J., Eggebeen J., Kitzman D.W. (2014). Skeletal muscle composition and its relation to exercise intolerance in older patients with heart failure and preserved ejection fraction. Am. J. Cardiol..

[54] Heinonen I., Bucci M., Kemppainen J., Knuuti J., Nuutila P., Boushel R., Kalliokoski K.K. (2012). Regulation of subcutaneous adipose tissue blood flow during exercise in humans. J. Appl. Physiol..

[55] Haykowsky M.J., Beaudry R., Brothers R.M., Nelson M.D., Sarma S., Gerche A.L. (2016). Pathophysiology of exercise intolerance in breast cancer survivors with preserved left ventricular ejection fraction. Clin. Sci..

[56] Didier K.D., Ederer A.K., Reiter L.K., Brown M., Hardy R., Caldwell J., Black C., Bemben M.G., Ade C.J. (2017). Altered blood flow response to small muscle mass exercise in cancer survivors treated with adjuvant therapy. J. Am. Heart Assoc..

[57] Schneider C.M., Hsieh C.C., Sprod L.K., Carter S.D., Hayward R. (2007). Cancer treatment-induced alterations in muscular fitness and quality of life: The role of exercise training. Ann. Oncol..

[58] Winters-Stone K.M., Bennett J.A., Nail L., Schwartz A. (2008). Strength, physical activity, and age predict fatigue in older breast cancer survivors. Oncol. Nurs. Forum..

[59] Kaya T., Karatepe A.G., Günaydn R., Yetiş H., Uslu A. (2010). Disability and health-related quality of life after breast cancer surgery: Relation to impairments. South Med. J..

[60] Butt Z., Rosenbloom S.K., Abernethy A.P., Beaumont J.L., Paul D., Hampton D., Jacobsen P.B., Syrjala K.L., Von Roenn J.H., Cella D. (2008). Fatigue is the most important symptom for advanced cancer patients who have had chemotherapy. JNCCN J. Natl. Compr. Cancer Netw..

[61] Villaseñor A., Ballard-Barbash R., Baumgartner K., Baumgartner R., Bernstein L., McTiernan A., Neuhouser M.L. (2012). Prevalence and prognostic effect of sarcopenia in breast cancer survivors: The HEAL Study. J. Cancer Surviv..

[62] Piraux E., Caty G., Reychler G. (2018). Effects of preoperative combined aerobic and resistance exercise training in cancer patients undergoing tumour resection surgery: A systematic review of randomised trials. Surg. Oncol..

[63] Pedersen B.K., Saltin B. (2015). Exercise as medicine—Evidence for prescribing exercise as therapy in 26 different chronic diseases. Scand J. Med. Sci. Sport..

[64] Liska D., Straska B., Pupis M. (2020). Physical Therapy as an Adjuvant Treatment for the Prevention and Treatment of Cancer. Klin. Onkol..

[65] Courneya K.S., Segal R.J., Mackey J.R., Gelmon K., Reid R.D., Friedenreich C.M., Ladha A.B., Proulx C., Vallance J.K.H., Lane K. (2007). Effects of aerobic and resistance exercise in breast cancer patients receiving adjuvant chemotherapy: A multicenter randomized controlled trial. J. Clin. Oncol..

[66] Schmitz K.H., Courneya K.S., Matthews C., Demark-Wahnefried W., Galvão D.A., Pinto B.M., Irwin M.L., Wolin K.Y., Segal R.J., Lucia A. (2010). American college of sports medicine roundtable on exercise guidelines for cancer survivors. Med. Sci. Sports Exerc..

[67] ACS Guidelines for Nutrition and Physical Activity [Internet]. https://www.cancer.org/healthy/eat-healthy-get-active/acs-guidelines-nutrition-physical-activity-cancer-prevention/guidelines.html.

[68] Hornsby W.E., Douglas P.S., West M.J., Kenjale A.A., Lane A.R., Schwitzer E.R., Ray K.A., Herndon J.E., Coan A., Gutierrez A. (2014). Safety and efficacy of aerobic training in operable breast cancer patients receiving neoadjuvant chemotherapy: A phase II randomized trial. Acta Oncol. (Madr.).

[69] Kolden G.G., Strauman T.J., Ward A., Kuta J., Woods T.E., Schneider K.L., Heerey E., Sanborn L., Burt C., Millbrandt L. (2002). A pilot study of group exercise training (GET) for women with primary breast cancer: Feasibility and health benefits. Psychooncology.

[70] Cheema B.S., Kilbreath S.L., Fahey P.P., Delaney G.P., Atlantis E. (2014). Safety and efficacy of progressive resistance training in breast cancer: A systematic review and meta-analysis. Breast Cancer Res. Treat..

[71] Speck R.M., Courneya K.S., Mâsse L.C., Duval S., Schmitz K.H. (2010). An update of controlled physical activity trials in cancer survivors: A systematic review and meta-analysis. J. Cancer Surviv..

[72] Khouri M.G., Hornsby W.E., Risum N., Velazquez E.J., Thomas S., Lane A., Scott J.M., Koelwyn G.J., Herndon J.E., Mackey J.R. (2014). Utility of 3-dimensional echocardiography, global longitudinal strain, and exercise stress echocardiography to detect cardiac dysfunction in breast cancer patients treated with doxorubicin-containing adjuvant therapy. Breast Cancer Res. Treat..

[73] Jones L.W., Liang Y., Pituskin E.N., Battaglini C.L., Scott J.M., Hornsby W.E., Haykowsky M. (2011). Effect of Exercise Training on Peak Oxygen Consumption in Patients with Cancer: A Meta-Analysis. Oncologist.

[74] Battaglini C., Bottaro M., Dennehy C., Rae L., Shields E., Kirk D., Hackney A. (2007). The effects of an individualized exercise intervention on body composition in breast cancer patients undergoing treatment. Sao Paulo Med. J..

[75] Jones L.W., Habel L.A., Weltzien E., Castillo A., Gupta D., Kroenke C.H., Kwan M.L., Quesenberry C.P., Scott J., Sternfeld B. (2016). Exercise and Risk of cardiovascular events in women with nonmetastatic breast cancer. J. Clin. Oncol..

[76] Palomo A., Ray R.M., Johnson L., Paskett E., Caan B., Jones L., Okwuosa T. (2017). Associations between exercise prior to and around the time of cancer diagnosis and subsequent cardiovascular events in women with breast cancer: A women’s ehalth initiative (WHI) analysis. J. Am. Coll. Cardiol..

[77] Ventura-Clapier R., Mettauer B., Bigard X. (2007). Beneficial effects of endurance training on cardiac and skeletal muscle energy metabolism in heart failure. Cardiovasc. Res..

[78] Fujimoto N., Prasad A., Hastings J.L., Arbab-Zadeh A., Bhella P.S., Shibata S., Palmer D., Levine B.D. (2010). Cardiovascular effects of 1 year of progressive and vigorous exercise training in previously sedentary individuals older than 65 years of age. Circulation.

[79] Haykowsky M.J., Mackey J.R., Thompson R.B., Jones L.W., Paterson D.I. (2009). Adjuvant trastuzumab induces ventricular remodeling despite aerobic exercise training. Clin. Cancer Res..

[80] Lee K., Tripathy D., Demark-Wahnefried W., Courneya K.S., Sami N., Bernstein L., Spicer D., Buchanan T.A., Mortimer J.E., Dieli-Conwright C.M. (2019). Effect of Aerobic and Resistance Exercise Intervention on Cardiovascular Disease Risk in Women with Early-Stage Breast Cancer: A Randomized Clinical Trial. JAMA Oncol..

[81] Kim C.J., Kang D.H., Smith B.A., Landers K.A. (2006). Cardiopulmonary responses and adherence to exercise in women newly diagnosed with breast cancer undergoing adjuvant therapy. Cancer Nurs..

[82] Scott J.M., Nilsen T.S., Gupta D., Jones L.W. (2018). Exercise therapy and cardiovascular toxicity in cancer. Circulation.

[83] Atella V., Piano Mortari A., Kopinska J., Belotti F., Lapi F., Cricelli C., Fontana L. (2019). Trends in age-related disease burden and healthcare utilization. Aging Cell.

[84] McLeod M., Breen L., Hamilton D.L., Philp A. (2016). Live strong and prosper: The importance of skeletal muscle strength for healthy ageing. Biogerontology.

[85] Battaglini C.L., Mills R.C., Phillips B.L., Lee J.T., Story C.E., Nascimento M.G.B., Hackney A.C. (2014). Twenty-five years of research on the effects of exercise training in breast cancer survivors: A systematic review of the literature. World J. Clin. Oncol..

[86] Hanson E.D., Wagoner C.W., Anderson T., Battaglini C.L. (2016). The Independent Effects of Strength Training in Cancer Survivors: A Systematic Review. Curr. Oncol. Rep..

[87] Hanson E.D., Srivatsan S.R., Agrawal S., Menon K.S., Delmonico M.J., Wang M.Q., Hurley B.F. (2009). Effects of strength training on physical function: Influence of power, strength, and body composition. J. Strength Cond. Res..

[88] Vincent K.R., Braith R.W., Feldman R.A., Kallas H.E., Lowenthal D.T. (2002). Improved cardiorespiratory endurance following 6 months of resistance exercise in elderly men and women. Arch. Intern Med..

[89] Frontera W.R., Meredith C.N., O’Reilly K.P., Evans W.J. (1990). Strength training and determinants of VO2(max) in older men. J. Appl. Physiol..

[90] Wagoner C.W., Hanson E.D., Ryan E.D., Brooks R., Wood W.A., Jensen B.C., Lee J.T., Coffman E.M., Battaglini C.L. (2019). Two weeks of lower body resistance training enhances cycling tolerability to improve precision of maximal cardiopulmonary exercise testing in sedentary middle-aged females. Appl. Physiol. Nutr. Metab..

[91] Dos Santos W.D.N., Gentil P., de Moraes R.F., Ferreira Júnior J.B., Campos M.H., de Lira C.A.B., Freitas Júnior R., Bottaro M., Vieira C.A. (2017). Chronic Effects of Resistance Training in Breast Cancer Survivors. Biomed. Res. Int..

[92] Rahnama N., Nouri R., Rahmaninia F., Damirchi A., Emami H. (2010). The effects of exercise training on maximum aerobic capacity, resting heart rate, blood pressure and anthropometric variables of postmenopausal women with breast cancer. J. Res. Med. Sci..

[93] Linschoten M., Teske A.J., Cramer M.J., van der Wall E., Asselbergs F.W. (2018). Chemotherapy-Related Cardiac Dysfunction: A Systematic Review of Genetic Variants Modulating Individual Risk. Circ. Genom. Precis. Med..

[94] Jones L.W., Hornsby W.E., Freedland S.J., Lane A., West M.J., Moul J.W., Ferrandino M.N., Allen J.D., Kenjale A.A., Thomas S.M. (2014). Effects of nonlinear aerobic training on erectile dysfunction and cardiovascular function following radical prostatectomy for clinically localized prostate cancer. Eur. Urol..

[95] Kirkham A.A., Bonsignore A., Bland K.A., McKenzie D.C., Gelmon K.A., Van Patten C.L., Campbell K.L. (2018). Exercise prescription and adherence for breast cancer: One size does not FITT All. Med. Sci. Sports Exerc..

[96] Quevedo-Jerez K., Gil-Rey E., Maldonado-Martín S., Herrero-Román F. (2019). Exercise-Intensity Adherence During Aerobic Training and Cardiovascular Response During Resistance Training in Cancer Survivors. J. Strength Cond. Res..

